# Intraventricular Hemorrhage and Survival, Multimorbidity, and Neurodevelopment

**DOI:** 10.1001/jamanetworkopen.2024.52883

**Published:** 2025-01-06

**Authors:** Philippa Rees, Chris Gale, Cheryl Battersby, Carrie Williams, Ben Carter, Alastair Sutcliffe

**Affiliations:** 1 Population Policy and Practice, Great Ormond Street UCL Institute of Child Health, London, United Kingdom; 2 Neonatal Medicine, School of Public Health, Faculty of Medicine, Imperial College London, London, United Kingdom; 3 Department of Biostatistics and Health Informatics, Institute of Psychiatry, Psychology & Neuroscience, King’s College London, United Kingdom

## Abstract

**Importance:**

Intraventricular hemorrhage (IVH) has proven to be a challenging and enduring complication of prematurity. However, its association with neurodevelopment across the spectrum of IVH severity, independent of prematurity, and in the context of contemporary care remains uncertain.

**Objective:**

To evaluate national trends in IVH diagnosis and the association with survival and neurodevelopmental outcomes at 2 years of age.

**Design, Setting, and Participants:**

This whole-population cohort study was conducted using data from the UK National Neonatal Research Database. Infants born at less than 29 weeks’ gestation with any grade of IVH between January 2013 and December 2019 in England were included and matched with controls. Data analysis occurred from November 2023 to June 2024.

**Exposure:**

IVH grades 1 to 4 (Papile classification).

**Main Outcomes and Measures:**

The primary outcome was survival without severe neurodevelopmental impairment (NDI) at 2 years’ corrected age including severe delays (inability to understand or use >5 words or signs; being unable to walk, sit, or use hands; blindness; or uncorrectable hearing impairment). Secondary outcomes included gross and fine motor function, receptive and expressive communication, vision, hearing, and overall developmental progress. Outcomes were derived from clinician-entered data and analyzed using multiple logistic regression.

**Results:**

Between 2013 and 2019, of 26 756 infants born at less 29 weeks’ gestation in England, 8461 received a diagnosis of IVH (5570 low-grade and 2891 high-grade, and 8328 were included in the study. Overall, 5519 included infants had low-grade IVH with a median [IQR] gestational age of 26 (25-27) weeks, of which 2477 (48.88%) were male. Of the 2809 included infants with high-grade IVH, the median (IQR) gestational age was 25 (24-26) weeks and 1710 (60.88%) were male. The mean (SD) incidence of high-grade IVH (108 [6.7] per 1000 live extremely preterm births) and low-grade IVH (208 [10.4] per 1000 live extremely preterm births) increased between 2013 and 2019, although this did not reach statistical significance for high-grade IVH. Survival without severe NDI decreased significantly after high-grade IVH (a 74% reduction; aOR, 0.26; 95% CI, 0.22-0.31), and to a lesser extent after low-grade IVH (a 12% reduction; aOR, 0.88; 95% CI, 0.79-0.98). Although low-grade IVH was associated with functional impairments, most survivors, 2283 of 4379 infants (52.15%), had no NDI, and the association with NDI was accounted for by grade 2 IVH. Decreased survival without severe NDI was observed with increasing grade of IVH, decreasing gestation, bilateral compared to unilateral injuries, and increasing morbidity count (severe retinopathy of prematurity, bronchopulmonary dysplasia, and surgical necrotizing enterocolitis). Impairments in gross motor function and communication were common, especially among those with high-grade IVH (with prevalences of 44.55% [715 of 1605 infants] and 48.91% [784 of 1603 infants], respectively).

**Conclusions and Relevance:**

In this cohort study, IVH was highlighted as a persistent issue with substantial neurodevelopmental implications despite advances in care. This study offers useful data for counseling families; however, follow-up to school age is necessary to grasp the full impact of these injuries on children’s lives.

## Key Points

**Question:**

Are advances in neonatal care associated with changes in rates of intraventricular hemorrhage (IVH), survival, or neurodevelopmental outcomes?

**Findings:**

This cohort study of 26 756 infants born at less than 29 weeks’ gestation demonstrated an increase in IVH rates between 2013 and 2019, although the increase in high-grade IVH rates did not reach statistical significance. High-grade IVH was associated with lower survival without severe neurodevelopmental impairment (a 74% reduction), as was low-grade IVH, but to a lesser extent (resulting in a 12% reduction).

**Meaning:**

These findings suggest that despite advances in neonatal care, IVH incidence is not declining, posing substantial challenges for neurodevelopment and emphasizing the need for ongoing monitoring and support for affected infants.

## Teaser

This cohort study analyzes associations of intraventricular hemorrhage with survival and neurodevelopmental outcomes among infants born at less than 29 weeks’ gestation in England.

## Introduction

Premature birth remains a critical public health issue, affecting approximately 10% of all live births in the UK and US annually.^1,2,3,4^ Infants born preterm face a higher risk of various complications and multimorbidity including intraventricular hemorrhage (IVH).^1,3^ IVH is a common complication of preterm birth, which can have profound implications for neurodevelopmental outcomes in affected infants.^1,5,6,7^

Clinical care for extremely preterm neonates has evolved considerably over the past 2 decades, with a view to reducing complications such as IVH and improving outcomes.^1,8^ Such evolutions include increased provision of antenatal steroids to prevent respiratory distress syndrome and antenatal magnesium sulfate for neuroprotection, a shift toward gentler and less invasive respiratory support, judicious surfactant administration, improved thermoregulation and nutrition, increased uptake of delayed cord clamping, and a drive toward earlier and longer kangaroo care.^1,8^ At an organizational level, care of children born extremely preterm has also been increasingly regionalized to facilitate delivery in tertiary neonatal units.^9,10^ These advances in neonatal care have led to improved survival among this population and reduced rates of IVH in some US centers.^10,11,12,13,14^ However, population estimates of the incidence of IVH, its association with additional morbidities of prematurity, and its impact on neurodevelopmental outcomes in the context of contemporary care remain uncertain.

High-grade IVH has historically been associated with an increased risk of neurodevelopmental impairment (NDI).^5,6^ However, the specific impact of low-grade IVH on development, beyond the challenges posed by prematurity and its associated complications, remains inadequately understood.^5,7^ Existing studies of the association of IVH with childhood outcomes involve relatively historical cohorts selected from specific neonatal centers or networks and many do not adequately account for the myriad of confounders that tend to accompany prematurity.^5,6,7^ Neonatal studies tend to focus heavily on neurodevelopmental assessment scores, such as the Bayley Scales of Infant and Toddler Development. However, these are rarely captured on a population-level, are poorly predictive of future childhood outcomes, and have limited utility when communicating with parents.^15,16,17,18^ Therefore, there is a pressing need for a whole population study of children born extremely preterm who received contemporary care to address these gaps. By examining functional outcomes following IVH compared with controls, this study aims to provide valuable insights into the distinct effects of IVH severity and the interplay of multimorbidity on functional neurodevelopment.

## Methods

### Ethics and Objective

This cohort study was approved by the Human Research Authority Research Ethics Committee and Confidential Advisory Group, with an exemption of informed consent due to or numerous reasons including that the data are held securely and pseudonomysed. The study is reported in line with the Strengthening the Reporting of Observational Studies in Epidemiology (STROBE) reporting guideline. The objective of the study was to evaluate trends in IVH diagnosis in extremely preterm infants and to assess the association of intraventricular hemorrhage with survival and neurodevelopmental outcomes at 2 years of age.

### Setting

Using the National Neonatal Research Database (NNRD), we captured neonatal data for our included population and their prospectively collected 2-year follow-up data. The NNRD contains patient-level demographic, clinical, and organizational data for all neonates admitted to any level of neonatal unit across Great Britain.^19,20^ These data (including maternal ethnicity) are derived from electronic patient records which are entered in real-time by clinicians.^19,21^ Submission of functional outcome data at 2 years is high (>87%) for surviving infants. These outcomes are ascertained at the 2-year clinical follow-up visit by trained health care professionals using a standardized national questionnaire focused on functional abilities.^22^

### Participants

All extremely preterm infants with any grade of IVH (1-4) identified on imaging born at less than 29 weeks’ gestation in England between 2013 and 2019 were included. All extremely preterm infants undergo regular cranial ultrasonography in UK neonatal units in the first days of life. Imaging is typically conducted and interpreted by clinicians. Both daily cranial ultrasonography data and episodic diagnoses of IVH using the Papile classification are captured by the NNRD.^23^ The highest recorded IVH grade was used, with grades 1 and 2 classified as low-grade and 3 and 4 as high-grade.

### Matched Controls

Infants with IVH were matched 1:1 to preterm infants without brain injury using Mahalanobis propensity score matching (to the nearest 20 neighbors) accounting for gestation, birthweight *z* score, sex, mode of delivery, multiple births, maternal smoking status, receipt of antenatal steroids, receipt of antenatal magnesium sulfate, and birth year.^24^ The nearest unique match was selected for each infant to minimize duplicate controls.

Because most IVH occurs in the first 24 hours of life, cases and controls who died in the first 24 hours were excluded from the 2-year outcome analyses. Data were extracted on co-occurring and evolving neurological injuries including cystic periventricular leukomalacia, posthemorrhagic ventricular dilatation, central nervous system infection, perinatal stroke, and seizures. Additional morbidities of interest included bronchopulmonary dysplasia (BPD), culture positive sepsis, surgically treated necrotizing enterocolitis, severe retinopathy of prematurity, and receipt of major surgery in the neonatal period (eTable 1 in Supplement 1).

### Outcome Measures

The primary outcome was survival without severe NDI at 2 years’ corrected age including severe (>12 months) developmental delay; inability to understand words or signs; inability to use more than 5 words or signs; being unable to walk, sit, or use hands; blindness; or uncorrectable hearing impairment (eTable 2 in Supplement 1).^22,25^ Secondary outcomes included gross and fine motor function, receptive and expressive communication, overall developmental progression, visual impairment, and hearing impairment (eTable 2 in Supplement 1).^25^

### Statistical Analysis

#### Primary Analysis

IVH incidence per 1000 preterm live births was plotted by birth year using Office for National Statistics live birth data. A linear regression model determined temporal trend in incidence with associated 95% CIs^26^; this was done for all infants with high- and low-grade IVH and a subgroup excluding those born at 22 to 23 weeks’ gestation.

Cases and controls were described and compared. A causal inference approach was employed, guided by a directed acyclic graph (DAG) specified a priori (before data access) to identify key covariates for matching and adjustment (eFigure 1 in Supplement 1).

Multiple logistic regression models estimated crude and adjusted odds ratios (aORs) with 95% CIs, adjusting for DAG-specified covariates. There are 12 neonatal operational delivery networks in England that coordinate care among tertiary and nontertiary neonatal units in each geographic region. Neonatal operational delivery network was included as a random effect in our regression models to account for heterogeneity between networks.^27^ The threshold for statistical significance was a 2-sided *P* < .05.

#### Handling Missing Data

Predictors of missing follow-up data were tested using forward selection (eFigure 2 in Supplement 1). Independent predictors of missing follow-up data and covariates from regression models were included in the multiple imputation models. These included brain injury presence, gestation, birthweight *z* score, birth year, maternal smoking in pregnancy, Index of Multiple Deprivation quintile (a geographically derived score of deprivation), multimorbidity presence, maternal age, level of discharging unit, number of unit admissions, number of previous pregnancies, sex, receipt of breastmilk at discharge, and admitting neonatal operational delivery network. Between 15 to 20 imputed datasets were generated. These datasets were combined using Rubin rules.^28^ The predominant mechanism of missingness was considered to be missing at random (MAR). However, the robustness of these models to deviations from the MAR assumption was tested using the delta method.^29^

Subgroup analyses determined survival without severe NDI by IVH grade, laterality, and week of gestation. The prevalence and impact of additional morbidities are presented. Among infants who survived to 36 weeks’ corrected gestation, the absolute risk of survival without severe NDI was determined in an additive model for those with no major morbidities, high-grade IVH alone, and high-grade IVH with 1 to 3 additional major morbidities.^30^ Prognostic independence was investigated for each major morbidity, and potential interactions between morbidities in the additive model were investigated and tested using χ^2^ tests.^30^ Analyses were conducted in Stata version 16 (StataCorp) and DAGs were created using the package Dagitty for R statistical software version 3.1 (R Project for Statistical Computing).^31^ Data analyses were conducted from November 2023 to June 2024.

## Results

Of the 26 756 infants live-born at less than 29 weeks’ gestation in England between 2013 and 2019, 8461 received a diagnosis of IVH and were included in incidence estimates (5570 with low-grade and 2891 with high-grade). Of these, 8328 infants (98.43%) with IVH surviving beyond 24 hours, and 7535 (89.06%) matched controls were included in outcomes analyses. The baseline characteristics are summarized in Table 1. Overall, 5519 infants with low-grade IVH were included (median [IQR] gestational age, 26 [25-27] weeks; 2477 [44.88%] male; mean [SD] maternal age, 30.8 [6.1] years); alongside 5027 matched controls (median [IQR] gestational age, 26 [25-27] weeks; 2834 [56.38%] male; mean [SD] maternal age, 30.5 [6.4] years). Additionally, 2809 infants with high-grade IVH were included (median [IQR] gestation age, 25 [24-26] weeks; 1710 [60.88%] male; mean [SD] maternal age, 30.3 [6.3] years) alongside 2508 matched controls (median [IQR] gestational age, 25 [24-26] weeks; 1403 [55.94%] male; mean [SD] maternal age, 30.3 [6.3] years). Primary outcome data were available for 12 497 included infants (78.78%) (eTable 3 in Supplement 1). Included infants were followed-up prospectively as part of the routine national follow-up program to April 2022.

**Table 1.** Baseline Characteristics of Those With High- and Low-Grade IVH and Their Matched Controls

Characteristic	Participants, No. (%) (N = 15 863)				Missing, No. (%) (N = 15 863)	Excluded, No./total No. (%) (n = 1059)^a^
	High-grade IVH (n = 2809)	Controls (n = 2508)	Low-grade IVH (n = 5519)	Controls (n = 5027)		
Maternal age, mean (SD), y	30.3 (6.3)	30.3 (6.3)	30.8 (6.1)	30.5 (6.4)	14 (0.1)	29.3 (6.2)
No. of previous pregnancies						
0	1044 (37.12)	886 (35.33)	2034 (36.85)	1833 (36.46)	NA	349/1059 (32.96)
1	669 (23.81)	637 (25.40)	1338 (24.24)	1248 (24.83)		267/1059 (25.21)
≥2	1097 (39.05)	985 (39.27)	2147 (38.90)	1946 (38.71)		443/1059 (41.83)
Maternal smoking	512 (18.23)	355 (14.15)	884 (16.02)	742 (14.76)	1746 (11.01)	177/1059 (21.71)
Index of Multiple Deprivation decile, mean (SD)	4.4 (2.7)	4.3 (2.8)	4.4 (2.8)	4.4 (2.8)	831 (5.24)	4.2 (2.8)
Maternal ethnicity						
Asian	289 (10.29)	281 (11.20)	608 (11.02)	544 (10.82)	2117 (13.35)	99/1059 (9.35)
Black	335 (11.93)	341 (13.60)	706 (12.79)	628 (12.49)		101/1059 (9.54)
Multiracial	34 (1.21)	25 (1.00)	87 (1.58)	65 (1.29)		4/1059 (0.38)
White	1630 (58.03)	1476 (58.85)	3174 (57.51)	3014 (59.96)		645/1059 (60.91)
Other^b^	47 (1.67)	32 (1.23)	86 (1.56)	70 (1.39)		12/1059 (1.13)
Receipt of antenatal steroids	2312 (82.31)	2161 (86.16)	4953 (89.74)	4443 (88.38)	199 (1.25)	613/995 (61.61)
Receipt of antenatal magnesium sulfate	1211 (43.11)^b^	891 (35.52)	2724 (49.36)	1730 (34.41)	5060 (31.90)	207/548 (37.77)
Receipt of surfactant	2441 (86.90)	2111 (84.17)	4376 (79.29)	4060 (80.76)	975 (6.12)	854/945 (90.37)
Birth year, mean (SD)	2016 (2.0)	2014 (3.4)	2016 (2.0)	2014 (3.4)	NA	2013 (3.3)
Gestational age, median (IQR), wk	25 (24-26)	25 (24-26)	26 (25-27)	26 (25-27)	NA	24 (23-25)
Birthweight, mean (SD), *z* score	−0.05 (0.8)	−0.13 (0.9)	−0.15 (0.9)	−0.19 (0.9)	NA	−0.09 (0.8)
Infant sex						
Female	1099 (39.12)	1066 (42.50)	2477 (44.88)	2125 (42.27)	104 (0.66)	462/1059 (43.62)
Male	1710 (60.88)	1403 (55.94)	3041 (55.12)	2834 (56.38)		570/1034 (55.13)
Multiple births	685 (24.39)	476 (18.98)	1284 (23.27)	1003 (19.95)	NA	244/1059 (23.04)
Receipt of breastmilk at discharge	1495 (53.22)	1533 (61.12)	3633 (65.83)	3146 (62.58)	NA	NA

Abbreviations: IVH, intraventricular hemorrhage; NA, not applicable.

^a^ Died within first 24 hours.

^b^ Other included Chinese or any ethnic group not otherwise specified.

### Incidence

**Figure 1.**
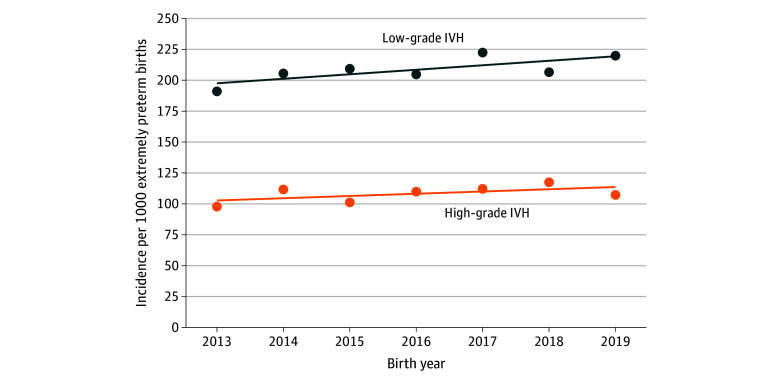
Trends and Incidence of High-Grade and Low-Grade Intraventricular Hemorrhage (IVH) for Children Born Extremely Preterm Between 2013 and 2019

The mean (SD) incidence of high-grade IVH was 108 (6.7) per 1000 live extremely preterm births births, and although there was an increase in incidence between 2013 and 2019, the difference was not statistically significant (*r* = 1.8; 95% CI −1.1 to 4.7). When excluding infants born at 22 to 23 weeks’ gestation, incidence also showed an increase, but the finding was not statistically significant (*r* = 2.8; 95% CI, −0.5 to 6.1) (Figure 1). The mean (SD) incidence of low-grade IVH was 208 (10.4) per 1000 live preterm births; this increased slightly during the study period (*r* = 3.6; 95% CI, 0.02 to 7.3). The increased incidence was attenuated when restricting to infants born at 24 to 28 weeks’ gestation (*r* = 2.2; 95% CI, −0.5 to 4.9) (Figure 1).

### Survival Without Severe NDI

Infants with high-grade IVH had a 74% reduction in survival without severe NDI compared with controls (aOR, 0.26; 95% CI, 0.22-0.31) (Table 2). The absolute crude risk of surviving without NDI was 32.77% (861 of 2627 infants) for those with a high-grade IVH compared with 59.78% (1342 of 2245 infants) for controls. Of those with high-grade IVH who survived, the subsequent absolute risk of severe NDI was 44.09% (709 of 1608 infants) compared with 19.65% of controls (342 of 1740 infants). Infants with low-grade IVH had a 12% reduction in survival without severe NDI compared with controls (aOR, 0.88; 95% CI, 0.79-0.98); however, the absolute risk difference was small (−0.78%) (Table 3). Most survivors with low-grade IVH (2283 of 4379 infants [52.15%]) had no NDI. Of 2627 infants with high-grade IVH and 5107 infants with low-grade IVH, 1766 (67.23%) and 1817 (35.58%) died or had severe NDI at 2 years’ corrected age.

**Table 2.** Survival and Neurodevelopmental Impairment After High-Grade IVH

Outcome	OR (95% CI)		Participants, No./total No. (%)		Imputed adjusted, OR (95% CI)^b^
	Crude	Adjusted^a^	Absolute imputed risk in high-grade IVH	Absolute imputed risk in controls	
Survival without severe NDI	0.33 (0.29-0.38)	0.25 (0.22-0.30)	861/2627 (32.77)	1342/2244 (59.78)	0.26 (0.22-0.31)
Outcomes for survivors					
Any NDI	3.09 (2.60-3.66)	3.54 (2.88-4.34)	1191/1609 (74.00)	815/1739 (46.89)	3.66 (2.93-4.58)
Severe NDI	3.11 (2.59-3.72)	3.59 (2.90-4.45)	709/1608 (44.09)	342/1741 (19.65)	3.68 (2.99-4.53)
Type of neurodevelopmental sequelae in survivors					
Overall development					
Mild delay (3-6 mo)	1.65 (1.31-2.07)	1.71 (1.30-2.24)	327/1139 (28.72)	302/1545 (19.54)	1.70 (1.25-2.32)
Moderate delay (6-12 mo)	2.65 (2,08-3.37)	2.87 (2.17-3.80)	347/1363 (25.45)	191/1671 (11.43)	2.74 (2.07-3.62)
Severe delay (>12 mo)	5.08 (3.77-6.84)	5.69 (4.01-8.08)	344/1603 (21.47)	88/1729 (5.09)	5.47 (3.91-7.65)
Communication					
Any difficulty with communication^^c^^	1.68 (1.43-1.99)	1.74 (1.43-2.12)	784/1603 (48.91)	629/1727 (36.40)	1.74 (1.42-2.14)
Difficulty with speech (<10 words or signs)	1.67 (1.41-1.98)	1.71 (1.41-2.09)	726/1601 (45.33)	580/1730 (33.54)	1.69 (1.42-2.03)
Difficulty understanding outside of familiar context	2.10 (1.64-2.71)	2.18 (1.63-2.93)	285/1596 (17.87)	165/1720 (9.59)	2.12 (1.60-2.80)
Has <5 meaningful words, vocalizations, or signs	1.95 (1.60-2.38)	2.24 (1.78-2.82)	474/1602 (29.58)	234/1375 (17.01)	2.30 (1.83-2.89)
Unable to understand words or signs	2.75 (1.96-3.85)	2.61 (1.79-3.82)	185/1596 (11.59)	84/1714 (4.90)	2.49 (1.67-3.69)
Motor					
Any difficulty walking	7.00 (5.62-8.71)	8.56 (6.42-10.87)	715/1605 (44.55)	175/1735 (10.09)	8.16 (6.23-10.67)
Unable to walk without assistance	7.24 (5.47-9.58)	9.28 (6.54-13.15)	467/1605 (29.11)	93/1738 (5.35)	8.55 (5.92-12.36)
Nonfluent or abnormal gait reducing mobility	7.16 (5.70-8.99)	8.78 (6.67-11.56)	676/1603 (42.18)	159/1738 (9.15)	8.34 (6.56-10.62)
Difficulty with the use of both hands	8.40 (4.95-14.25)	9.47 (4.99-17.98)	167/1604 (10.41)	25/1678 (1.49)	8.48 (4.30-16.74)
Difficulty with using 1 hand	16.54 (10.81-25.31)	18.84(11.53-30.79)	419/1603 (26.14)	36/1748 (2.06)	18.51 (11.52-29.72)
Unstable or needs support sitting	11.34 (6.62-19.43)	12.83 (6.84-24.05)	204/1604 (12.72)	22/1658 (1.327)	12.29 (6.55-23.07)
Unable to sit	9.35 (4.98-17.56)	9.35 (4.62-18.91)	130/1608 (8.09)	19/1743 (1.09)	7.94 (4.13-15.29)
Unable to use hands (eg, to feed)	9.07 (5.35-15.36)	10.36 (5.63-19.06)	179/159 (11.20)	25/1748 (1.43)	9.75 (5.23-18.16)
Vision					
Any vision problems including squint	3.37 (2.71-4.19)	3.94 (3.05-5.10)	504/1595 (31.61)	202/1725 (11.71)	4.02 (3.07-5.27)
A not fully correctable visual defect	4.98 (3.25-7.64)	6.10 (3.71-10.03)	175/1583 (11.05)	42/1714 (2.45)	5.82 (3.39-9.43)
Complete blindness or able to see light only	NA	NA	NA	NA	NA
Auditory					
Any hearing impairment	2.37 (1.63-3.43)	2.48 (1.56-3.93)	131/1600 (8.19)	65/1715 (3.79)	2.20 (1.47-3.29)
Hearing impairment correctable with aids^d^	1.53 (0.86-2.71)	1.32 (0.63-2.79)	39/1625 (2.40)	30/1786 (1.68)	1.29 (0.61-2.72)
Hearing impairment not correctable with aids	4.04 (1.64-9.97)	5.17 (1.50-17.86)	40/1587 (2.52)	11/1746 (0.63)	4.37 (1.25-15.25)

Abbreviations: IVH, intraventricular hemorrhage; NDI, neurodevelopmental impairment; OR, odds ratio.

^a^ Adjusted for gestation, birthweight *z* score, antenatal steroids, sex, Index of Multiple Deprivation quintile, smoking in pregnancy, and admission neonatal operational delivery network fitted as a random effect.

^b^ Predictors of missingness used in imputed models included maternal age, Index of Multiple Deprivation quintile, birth year, gestation weeks, birthweight *z *score, multimorbidity, smoking in pregnancy, discharge unit level, number of previous pregnancies, breastmilk at discharge, and admission neonatal operational delivery network.

^c^ Multimorbidity and breastmilk at discharge were not included as predictors of missingness because the model failed to converge, and they did not predict missingness for this specific outcome. Neonatal operational delivery network not fitted as random effect due to insufficient numbers.

^d^ Index of Multiple Deprivation quintile was not included as a predictor because it was perfect predictor of the outcome (ie, missingness).

**Table 3.** Survival and Neurodevelopmental Impairment After Low-Grade IVH

Outcome	OR (95% CI)		Participants, No./total No. (%)		Imputed adjusted, OR (95% CI)^b^
	Crude	Adjusted^a^	Absolute imputed risk in low-grade IVH	Absolute imputed risk in controls	
Survival without severe NDI	1.00 (0.91-1.10)	0.90 (0.80-1.01)	3290/5107 (64.41)	2908/4462 (65.19)	0.88 (0.79-0.98)
Outcomes for survivors					
Any NDI	1.13 (1.00-1.25)	1.16 (1.03-1.31)	2096/4378 (47.86)	1659/3706 (44.77)	1.17 (1.04-1.32)
Severe NDI	1.25 (1.10-1.42)	1.32 (1.14-1.52)	996/4378 (22.75)	707/3704 (19.09)	1.29 (1.11-1.49)
Type of neurodevelopmental sequelae in survivors					
Overall development					
Mild delay (3-6 mo)	1.06 (0.91-1.23)	1.08 (0.91-1.28)	683/3755 (18.19)	562/3272 (17.18)	1.09 (0.91-1.31)
Moderate delay (6-12 mo)	1.16 (0.98-1.38)	1.14 (0.94-1.39)	507/4102 (12.36)	386/3748 (10.30)	1.14 (0.94-1.39)
Severe delay (>12 mo)	1.30 (1.06-1.61)	1.36 (1.07-1.73)	339/4346 (7.80)	221/3683 (6.00)	1.38 (1.10-1.73)
Communication					
Any difficulty with communication	1.09 (0.97-1.21)	1.08 (0.94-1.22)	1522/4353 (34.95)	1215/3689 (32.93)	1.10 (0.96-1.26)
Difficulty with speech (<10 words or signs)	1.17 (1.04-1.31)	1.15 (1.01-1.32)	1432/4346 (32.95)	1095/3680 (29.74)	1.16 (1.01-1.33)
Difficulty understanding outside of familiar context	1.17 (0.97-1.41)	1.03 (0.83-1.29)	425/4345 (9.78)	321/3673 (8.74)	1.06 (0.86-1.29)
Has <5 meaningful words, vocalizations, or signs	1.22 (1.06-1.41)	1.19 (1.01-1.41)	759/4337 (17.50)	549/3683 (14.91)	1.20 (1.03-1.41)
Unable to understand words or signs	1.40 (1.10-1.80)	1.39 (1.03-1.86)	248/4340 (5.72)	157/3659 (4.29)	1.34 (0.98-1.85)
Motor					
Any difficulty walking	1.17 (1.0-1.37)	1.31 (1.09-1.57)	557/4365 (12.76)	402/3700 (10.87)	1.30 (1.07-1.59)
Unable to walk without assistance	1.42 (1.15-1.75)	1.53 (1.20-1.95)	349/4363 (8.00)	211/3688 (5.72)	1.51 (1.21-1.89)
Nonfluent or abnormal gait reducing mobility	1.21 (1.02-1.43)	1.36 (1.12-1.65)	514/4368 (11.77)	356/3694 (9.64)	1.37 (1.12-1.66)
Difficulty with the use of both hands	1.52 (1.02-2.25)	1.61 (1.01-2.57)	102/4359 (2.34)	57/3701 (1.54)	1.61 (0.97-2.65)
Difficulty with using 1 hand	1.18 (0.85-1.63)	1.20 (0.81-1.78)	126/4360 (2.89)	91/3699 (2.46)	1.20 (0.81-1.77)
Unstable or needs support sitting	1.64 (1.15-2.35)	1.67 (1.10-2.54)	130/4377 (2.97)	72/3711 (1.94)	1.56 (1.01-2.42)
Unable to sit	2.11 (1.28-3.46)	2.56 (1.37-4.81)	82/4362 (1.88)	31/3690 (0.84)	2.65 (1.36-5.16)
Unable to use hands (eg, to feed)^c^	1.34 (0.95-1.88)	1.20 (0.82-1.76)	127/4360 (2.91)	85/3696 (2.30)	1.19 (0.83-1.70)
Vision					
Any vision problems including squint^c^	1.45 (1.22-1.71)	1.49 (1.23-1.81)	571/4343 (13.15)	351/3685 (9.52)	1.48 (1.21-1.81)
A not fully correctable visual defect	1.49 (0.98-2.26)	1.41 (0.88-2.24)	98/4318 (2.27)	61/3653 (1.67)	1.30 (0.82-2.08)
Complete blindness or able to see light only^d^	1.50 (0.55-4.06)	1.32 (0.38-4.57)	20/4348 (0.46)	16/382 (4.19)	1.01 (0.26-3.87)
Hearing					
Any hearing impairment	0.86 (0.66-1.11)	0.99 (0.73-1.35)	176/4439 (4.06)	176/3682 (4.78)	0.92 (0.66-1.29)
Hearing impairment correctable with aids	0.66 (0.45-0.97)	0.81 (0.51-1.29)	69/4313 (1.60)	85/3696 (2.30)	0.79 (0.49-1.27)
Hearing impairment not correctable with aids	0.75 (0.41-1.35)	0.90 (0.45-1.79)	34/4304 (0.79)	38/3725 (1.02)	0.87 (0.47-1.61)

Abbreviations: IVH, intraventricular hemorrhage; NDI, neurodevelopmental impairment; OR, odds ratio.

^a^ Adjusted for gestation, birthweight *z* score, Index of Multiple Deprivation quintile, smoking in pregnancy, sex, and admitting neonatal operational delivery network fitted as a random effect.

^b^ Predictors of missingness included low-grade IVH, gestation, birthweight *z* score, birth year, maternal smoking, Index of Multiple Deprivation quintile, multimorbidity, maternal age, discharging unit level, neonatal unit count, number of previous pregnancies, sex, and admitting neonatal operational delivery network.

^c^ Multimorbidity was not included as a predictor of missingness due to failure to converge.

^d^ Multimorbidity and discharging unit level were not included as predictor of missingness.

**Figure 2.**
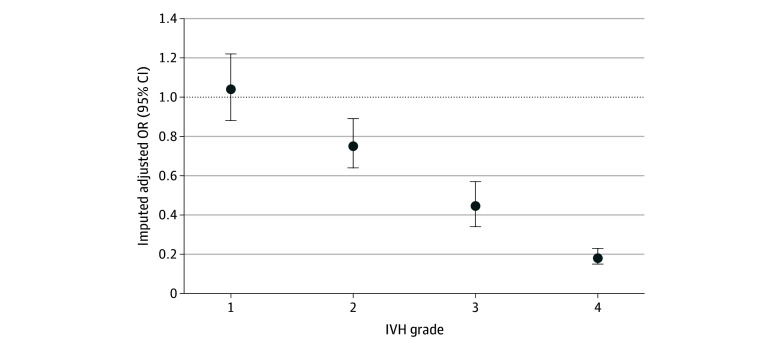
The Likelihood of Survival Without Severe Neurodevelopmental Impairment by Each Grade of Intraventricular Hemorrhage (IVH) OR indicates odds ratio.

There was stepwise reduction in the likelihood of surviving without severe NDI by each grade of IVH (Figure 2); there was no difference for grade 1 IVH (aOR, 1.04; 95% CI, 0.88-1.22), a 25% reduction after grade 2 IVH (aOR, 0.75; 95% CI, 0.64-0.89), a 56% reduction after grade 3 IVH (aOR, 0.44; 95% CI, 0.34-0.57), and an 82% reduction after grade 4 IVH (aOR, 0.18; 95% CI, 0.15-0.23) (eTable 4 in Supplement 1). Laterality data were only available for 75.40% of those with high-grade IVH (2118 of 2809 infants) and 70.39% with low-grade IVH (3885 of 5519 infants). The absolute risk of surviving without severe NDI was 70.58% (1037 of 1469 infants) for those with unilateral low-grade IVH compared with 60.49% (910 of 1504 infants) for those with bilateral low-grade IVH. The absolute risk of surviving without severe NDI was 54.52% (130 of 238 infants) for those with unilateral high-grade IVH compared with 33.43% (421 of 1259 infants) for those with bilateral high-grade IVH (eTable 4 in Supplement 1). There was also a trend toward an increasing absolute risk of surviving without severe NDI with each additional week of gestation, from 12.81% (51 of 398 infants) at 23 weeks’ gestation to 44.51% (122 of 274 infants) at 28 weeks’ gestation for those with high-grade IVH (eTable 5 in Supplement 1).

Most infants surviving to 36 weeks’ corrected age had additional morbidities; BPD was particularly common in cases and controls. Of those with high-grade IVH, 13.03% (366 of 2809 infants) developed posthemorrhagic ventricular dilatation, 4.70% (132 of 2809 infants) had a ventriculoperitoneal shunt inserted, and 11.78% (331 of 2809 infants) went on to develop cystic periventricular leukomalacia (eTable 6 in Supplement 1). Each major morbidity (high-grade IVH, BPD, severe retinopathy of prematurity, and surgical necrotizing enterocolitis) significantly and independently reduced the likelihood of survival without severe NDI and there were no significant interactions between morbidity pairs. In those with high-grade IVH, there was a linear decrease in the proportion surviving without severe NDI with each additional major morbidity: 82.99% in those with no IVH or major morbidities (653 of 787 infants); 59.36% in those with high-grade IVH alone (595 of 1002 infants); and 62.62% (1814 of 2897 infants), 35.51% (181 of 510 infants), and 24.4% (12 of 49 infants) in those with high-grade IVH and 1 to 3 additional major morbidities, respectively (eFigure 3 in Supplement 1). Sensitivity analyses demonstrated that multiple imputation models were robust to moderate deviations from the MAR assumption, with minimal changes in effect size estimates even in the most extreme MNAR scenarios modeled (eTable 7 and eTable 8 in Supplement 1).

### Neurodevelopmental Outcomes Among Survivors

#### Developmental Delay

Compared with controls, survivors of high-grade IVH had 70% increased odds of mild developmental delay (aOR, 1.70; 95% CI, 1.25-2.32), a 174% increase in odds of moderate developmental delay (aOR, 2.74; 95% CI, 2.07-3.62), and more than 5 times the odds of severe developmental delay (aOR, 5.47; 95% CI, 3.91-7.65) (Table 2). Survivors of low-grade IVH had an increased risk of severe developmental delay (aOR, 1.38; 95% CI, 1.10-1.73) (Table 3). Importantly, the majority of survivors of low-grade IVH had no developmental delay (2051 of 3186 infants [64.38%]).

#### Gross and Fine Motor

Surviving infants with high-grade IVH had a substantially increased risk of both gross and fine motor impairments at 2 years’ corrected age across the spectrum of severity (Table 2). Almost one-half of children with a high-grade IVH (715 of 1605 infants [44.55%]) had some difficulty walking compared with 10.09% of controls (175 of 1734 infants; aOR, 8.16; 95% CI, 6.23-10.67). Gross motor impairments were more prevalent than fine motor impairments.

Surviving infants with low-grade IVH also had an increased risk of gross motor impairments compared with controls across the spectrum of severity. However, this was far less prevalent than in high-grade IVH, and the absolute risk difference compared with controls was low; only 12.76% (557 of 4365 infants) had difficulty walking compared with 10.87% of controls (402 of 3698 infants; aOR, 1.30; 95% CI, 1.07-1.59) (Table 3). Survivors of low-grade IVH did not have increased risks of fine motor impairments.

#### Communication

Although difficulty with communication was common in both the high-grade IVH group (784 of 1603 infants [48.91%]) and controls (629 of 1728 infants [36.40%]), there was a relatively increased risk after high-grade IVH (aOR, 1.74; 95% CI, 1.42-2.14). Increased risks of both receptive and expressive communication impairments across the spectrum of severity were seen after high-grade IVH (Table 2). Infants with low-grade IVH had increased risks of some expressive communication impairments but not receptive impairments; however, the absolute risk difference between those with low-grade IVH and controls was small (Table 3).

#### Vision

Infants with high-grade IVH had increased risks of any visual impairment (aOR, 4.02; 95% CI, 3.07-5.27) and visual impairment that was not fully correctable (aOR, 5.82; 95% CI, 3.39-9.43) compared with controls, although most surviving infants with high-grade IVH did not have any visual impairment (1090 of 1594 infants [68.38%]). Fortunately, complete blindness was rare (Table 2). Infants with low-grade IVH had an increased risk of any visual impairment (aOR, 1.48; 95% CI, 1.21-1.81), but the absolute risk was small (571 of 4342 infants [13.15%]), and they did not have increased risks of more severe visual impairments (Table 3).

#### Hearing

Increased risks of any hearing impairment and uncorrectable hearing impairments were seen after high-grade IVH, although these were rare (Table 2). No increased risks of hearing impairments were seen after low-grade IVH (Table 3).

## Discussion

This cohort study reports that the incidence of low-grade IVH increased in the UK over the last decade. The incidence of high-grade IVH also increased during this time although this did not reach statistical significance. The importance of this is reiterated by the finding that 67.2% of infants with high-grade IVH and 35.6% with low-grade IVH died or had severe NDI at 2 years’ corrected age. Lower survival without severe NDI was seen by increasing grade of IVH, bilateral compared with unilateral IVH, decreasing gestational age, and with increasing numbers of additional major morbidities. Gross motor and expressive communication impairments were prevalent.

The national incidence of high- and low-grade IVH reported in this study (10.8% and 20.8%, respectively) is consistent with international estimates.^1,32,33^ The increasing incidence is also mirrored by the Australian New Zealand Neonatal Network.^34,35^ Additionally, although the incidence of severe IVH across the 19 US academic centers included in the Eunice Kennedy Shriver National Institute of Child Health and Human Development Neonatal Research Network and the Canadian Neonatal Network has shown modest reductions, these have also started to plateau.^1,32,33^

Further investigation into the underlying reasons for the persistent rates of IVH is required to determine whether they are due to changes in the underlying population or changes in care. This is particularly pressing given that active resuscitation is now widely offered to infants born at 22 to 23 weeks’ gestation with the greatest risk of IVH.^36,37^

This study provides further insight into the conflicting evidence on the impact of low-grade IVH on neurodevelopment. While our findings corroborate a recent meta-analysis^5^ reporting increased risks of moderate to severe NDI following low-grade IVH, the difference in absolute risks compared with controls was small and unlikely to be clinically significant. Due to the size of this study, we were able to demonstrate this effect was due to the higher incidence of adverse outcomes among those with grade 2 IVH. Previous studies were not powered to investigate this.^27^ The significant decrease in survival without severe NDI following high-grade IVH, and the additive negative association of morbidity count is also consistent with historical studies.^5,30^ The breakdown of these risks by grade of IVH, laterality, week of gestation, and additional morbidities in a contemporary population cohort provides valuable prognostic data for use when counseling families during their neonatal unit journey. These data specifically support counseling families that grade 1 IVH does not appear to increase the risk of death or neurodevelopmental sequelae.

### Limitations

While this study benefitted from the use of whole population data, rendering it more generalizable and less susceptible to selection bias, it did have several limitations. IVH was determined on cranial ultrasonography conducted as part of routine clinical care by neonatologists, and although reasonable interrater reliability has been demonstrated in identifying high-grade IVH, it may be poorer for lower IVH grades.^38,39^ This imaging modality also does not reliably identify cerebellar or white matter injury.^39^ Magnetic resonance imaging is not routinely offered to this population; therefore, the impact of additional and potentially confounding injuries could not be explored. We did not have data on withdrawal of intensive care which could have impacted mortality estimates for a small number of the most preterm infants and those with particularly severe IVH. Additionally, caution is warranted in interpreting the association of IVH with NDI due to their association with additional neonatal morbidities.

Multiple imputation was undertaken to account for missing follow-up data, which demonstrated predictable missingness. These methods have been widely employed and validated by other preterm follow-up studies.^29,40,41,42^ Reassuringly, effect size estimates did not differ between the complete case analyses and the imputed models, and sensitivity analyses confirmed that these models were robust to modest departures from the MAR assumption.

Assessment of outcomes at 2 years has limitations; these are not long-term outcomes and are not necessarily fixed or predictive of later outcomes.^15,43^ Further follow-up of this cohort to school age is therefore vital.

## Conclusions

In this cohort study of children born extremely preterm in the UK between 2013 and 2019, the incidence of low- and high-grade IVH showed an increasing trend. Both high- and low-grade IVH were associated with reduced survival without severe NDI, with a minimal impact observed following low-grade IVH and no discernible effect noted after grade 1 IVH. This study offers key data for family counseling. Follow-up to school age is essential to fully understand the long-term impact of these injuries on children’s lives.

## Notes

Supplement 1. **eTable 1.** Definitions of Comorbidities **eTable 2.** Outcome Definitions **eFigure 1.** Directed Acyclic Graph for Causal Pathway Between IVH and Death or Neurodevelopmental Impairment **eFigure 2.** Directed Acyclic Graph of Causal Pathways for Missing Outcome Data **eTable 3.** Missing Data for Cases and Control by Outcome **eTable 4.** Survival Without Severe Neurodevelopmental Impairment by Each Grade of IVH and Laterality of Injury **eTable 5.** Survival Without Severe Neurodevelopmental Impairment by Each Week of Gestation After High-Grade IVH **eTable 6.** Table Illustrating the Prevalence of Multimorbidity for Those With High and Low-Grade IVH Compared to Controls **eFigure 3.** Graph Illustrating the Proportion of Infants Surviving Without Severe Neurodevelopmental Impairment by Increasing Morbidity Count **eTable 7.** Estimated Risk of Survival Without Severe Neurodevelopmental Impairment Under Different Missing at Random (MAR) and Missing Not at Random (MNAR) Scenarios **eTable 8.** Absolute Imputed Risk of Survival Without Severe Neurodevelopmental Impairment Under Missing At Random (MAR) Assumption and a Moderate Missing Not at Random (MNAR) Scenario

Supplement 2.
Data Sharing Statement
