# Supplementary material for: Intraventricular Hemorrhage and Survival, Multimorbidity, and Neurodevelopment
Source: JAMA Netw Open. 2025 Jan 6;8(1):e2452883. doi: 10.1001/jamanetworkopen.2024.52883 (PMC11704976; doi:10.1001/jamanetworkopen.2024.52883)
Supplement: Supplement 1. — eTable 1. Definitions of Comorbidities eTable 2. Outcome Definitions eFigure 1. Directed Acyclic Graph for Causal Pathway Between IVH and Death or Neurodevelopmental Impairment eFigure 2. Directed Acyclic Graph of Causal Pathways for Missing Outcome Data eTable 3. Missing Data for Cases and Control by Outcome eTable 4. Survival Without Severe Neurodevelopmental Impairment by Each Grade of IVH and Laterality of Injury eTable 5. Survival Without Severe Neurodevelopmental Impairment by Each Week of Gestation After High-Grade IVH eTable 6. Table Illustrating the Prevalence of Multimorbidity for Those With High and Low-Grade IVH Compared to Controls eFigure 3. Graph Illustrating the Proportion of Infants Surviving Without Severe Neurodevelopmental Impairment by Increasing Morbidity Count eTable 7. Estimated Risk of Survival Without Severe Neurodevelopmental Impairment Under Different Missing at Random (MAR) and Missing Not at Random (MNAR) Scenarios eTable 8. Absolute Imputed Risk of Survival Without Severe Neurodevelopmental Impairment Under Missing At Random (MAR) Assumption and a Moderate Missing Not at Random (MNAR) Scenario [file jamanetwopen-e2452883-s001.pdf]

Supplemental Online Content

Rees P, Gale C, Battersby C, Williams C, Carter B, Sutcliffe A. Intraventricular hemorrhage and survival, multimorbidity, and neurodevelopment. *JAMA Netw Open*. 2025;8(1):e2452883. doi:10.1001/jamanetworkopen.2024.52883

eTable1. Definitions of Comorbidities
eTable 2. Outcome Definitions
eFigure 1. Directed Acyclic Graph for Causal Pathway Between IVH and Death or Neurodevelopmental Impairment
eFigure 2. Directed Acyclic Graph of Causal Pathways for Missing Outcome Data
eTable 3. Missing Data for Cases and Control by Outcome
eTable 4. Survival Without Severe Neurodevelopmental Impairment by Each Grade of IVH and Laterality of Injury
eTable 5. Survival Without Severe Neurodevelopmental Impairment by Each Week of Gestation After High-Grade IVH
eTable 6. Table Illustrating the Prevalence of Multimorbidity for Those With High and Low-Grade IVH Compared to Controls
eFigure 3. Graph Illustrating the Proportion of Infants Surviving Without Severe Neurodevelopmental Impairment by Increasing Morbidity Count
eTable 7. Estimated Risk of Survival Without Severe Neurodevelopmental Impairment Under Different Missing at Random (MAR) and Missing Not at Random (MNAR) Scenarios
eTable 8. Absolute Imputed Risk of Survival Without Severe Neurodevelopmental Impairment Under Missing At Random (MAR) Assumption and a Moderate Missing Not at Random (MNAR) Scenario

This supplemental material has been provided by the authors to give readers additional information about their work.

© 2024 Rees P et al. *JAMA Network Open.*

eTable 1: definitions of additional morbidities	
Morbidity	Definition
Bronchopulmonary dysplasia	Receipt of respiratory support at 36 weeks corrected gestational age.
Severe retinopathy of prematurity	Bilateral or unilateral retinopathy of prematurity grade 4, 5 or aggressive posterior.
Surgical necrotizing enterocolitis	Receipt of surgical treatment for necrotizing enterocolitis (on daily data).
Culture-positive sepsis	Confirmed gram positive sepsis, gram negative sepsis, bacterial meningitis or fungal infection on daily data (at any point) or a positive blood or CSF culture.
Major surgery	Any major surgery (on daily data) defined as entry into any body cavity, or any surgery with a large volume of anticipated blood loss relative to the infant size.

© 2024 Rees P et al. *JAMA Network Open*.

eTable 2: Functional outcomes assessed and the corresponding developmental domains (* considered a severe neurodevelopmental impairment)[27]

Domain	Questions
Developmental delay	Is the child’s development between 3-6 months behind corrected age?
	Is the child’s development between 6-12 months behind corrected age?
	*Is the child’s development more than 12 months behind corrected age?
Receptive communication	Does this child have difficulty with understanding outside of familiar context?
	*Is this child unable to understand words or signs?
Expressive communication	Does this child have any difficulty with communication?
	Does this child have difficulty with speech (<10 words/signs)?
	*Does the child have <5 meaningful words, vocalization or signs?
Gross motor	Does this child have any difficulty walking?
	Is this child’s gait non-fluent or abnormal reducing mobility?
	*Is this child unable to walk without assistance?
	Is this child unstable or needs to be supported when sitting?
	*Is this child unable to sit?
Fine motor	Does this child have any difficulty with the use of one hand?
	Does this child have difficulty with the use of both hands?
	*Is this child unable to use hands (i.e. to feed)?
Vision	Does this child have any visual problems (including squint)?
	Does this child have a visual defect that is not fully correctable?
	*Is this child blind or sees only light?
Hearing	Does this child have a hearing impairment?
	Does this child have a hearing impairment corrected by aids?
	*Does this child have hearing impairment not correctable with aids?

© 2024 Rees P et al. *JAMA Network Open*.

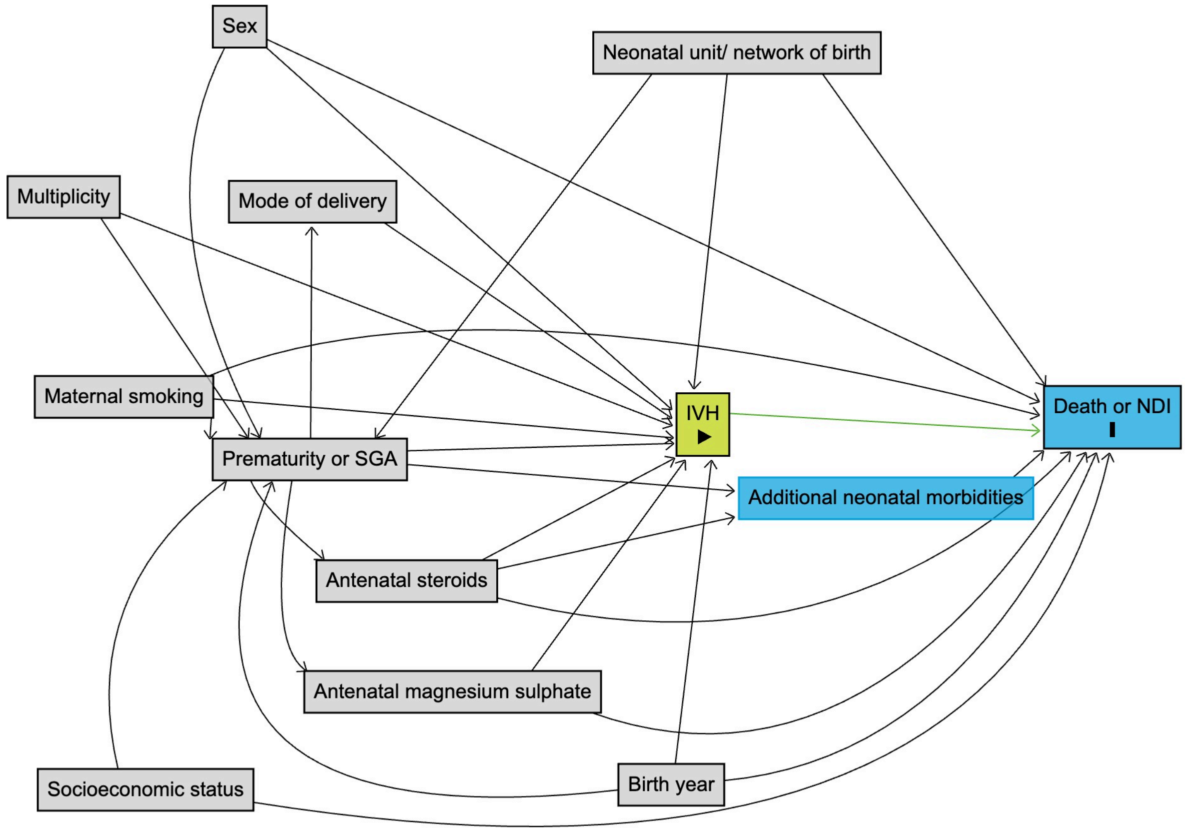

eFigure 1: Directed acyclic graph illustrating assumed causal relationship between intraventricular hemorrhage and death or neurodevelopmental impairment. Additional neonatal morbidities include retinopathy of prematurity, necrotizing enterocolitis, bronchopulmonary dysplasia, and neonatal sepsis.

© 2024 Rees P et al. *JAMA Network Open*.

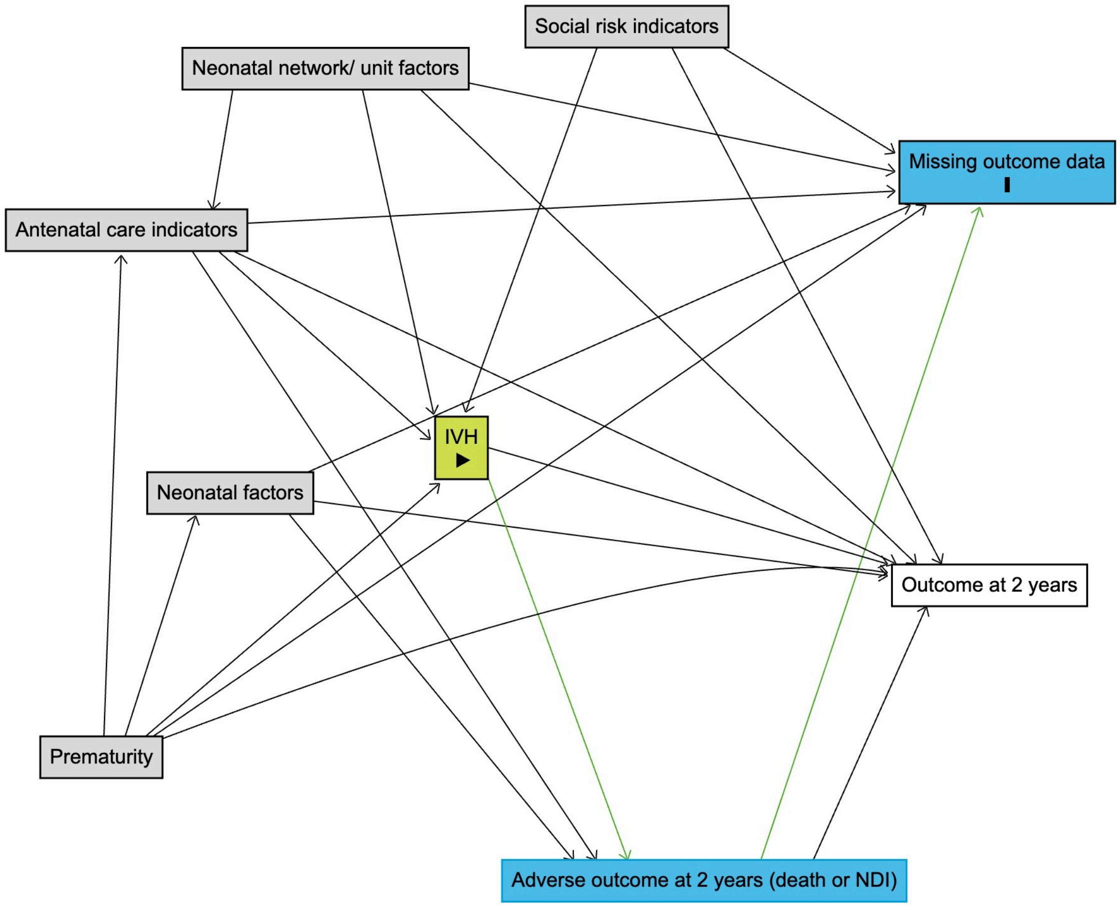

eFigure 2: Directed acyclic graph depicting the assumptions made about the mechanisms of missingness for 2-year outcome data. Antenatal care indicators included administration of magnesium sulphate, antenatal steroids and mode of delivery. Neonatal factors included sex, bronchopulmonary dysplasia, retinopathy of prematurity, necrotizing enterocolitis, major surgery, sepsis, and multiplicity. Social risk indicators included number of previous pregnancies, maternal age, maternal ethnicity, smoking in pregnancy, receipt of breast milk at discharge and index of median deprivation. Neonatal network/ unit factors included the admitting neonatal network, the number of neonatal unit admissions per infant (i.e. transfers), and level of discharging neonatal unit.

© 2024 Rees P et al. *JAMA Network Open*.

eTable 3: Missing data for cases and controls by outcome								
Outcome	High-grade IVH, n (%)	Missing outcome data, n (%)	Controls, n (%)	Missing outcome data, n (%)	Low-grade IVH, n (%)	Missing outcome data, n (%)	Controls, n (%)	Missing outcome data, n (%)
Survival without severe NDI	670 (30.28)	596 (21.22)	979 (56.56)	777 (30.98)	2,507 (63.09)	1,539 (27.89)	2,161 (63.08)	1,601 (31.85)
Outcomes for survivors								
Severe NDI	523 (43.84)	596 (33.31)	246 (20.08)	777 (38.81)	737 (22.72)	1,545 (32.26)	509 (19.06)	1,601 (37.49)
Overall development								
Any delay	662 (57.27)	633 (35.38)	385 (32.06)	801 (40.01)	1,135 (35.12)	1,557 (32.51)	852 (32.01)	1,609 (37.67)
Communication								
Any difficulty with communication	563 (48.79)	635 (35.49)	430 (36.13)	812 (40.56)	1,099 (34.87)	1,637 (34.18)	849 (33.02)	1,700 (39.80)
Difficulty with speech (<10 words or signs)	522 (45.51)	642 (35.89)	395 (33.33)	817 (40.81)	1,037 (33.05)	1,651 (34.47)	757 (29.7)	1,722 (40.32)
Difficulty understanding outside of familiar context	196 (17.71)	682 (38.12)	107 (9.28)	849 (42.41)	302 (9.81)	1,709 (35.69)	212 (8.49)	1,775 (41.56)
Has <5 meaningful words, vocalizations or signs	334 (29.20)	645 (36.05)	204 (17.42)	831 (41.51)	549 (17.60)	1,669 (34.85)	378 (14.85)	1,726 (40.41)
Unable to understand words or signs	124 (11.26)	688 (38.46)	51 (4.41)	846 (42.26)	177 (5.75)	1,711 (35,73)	104 (4.16)	1,773 (41.51)
Motor								
Any difficulty walking	521 (44.19)	610 (34.10)	123 (10.17)	792 (39.56)	406 (12.66)	1,583 (33.05)	291 (11.02)	1,630 (38.16)
Unable to walk without assistance	341 (29.25)	623 (34.82)	65 (5.40)	798 (39.86)	256 (8.01)	1,593 (33.26)	152 (5.78)	1,642 (38.45)
Non-fluent or abnormal gait reducing mobility	488 (41.85)	623 (34.82)	110 (9.14)	798 (39.86)	372 (11.65)	1,596 (33.33)	259 (9.84)	1,638 (38.35)
Difficulty with the use of both hands	118 (10.23)	635 (35.49)	16 (1.34)	806 (40.26)	72 (2.26)	1,609 (33.60)	39 (1.50)	1,676 (39.24)
Difficulty with using one hand	293 (25.37)	634 (35.44)	24 (2.01)	810 (40.46)	90 (2.84)	1,619 (33.81)	63 (2.42)	1,673 (39.17)
Unstable or needs support sitting	146 (12.58)	628 (35.10)	15 (1.25)	804 (40.16)	91 (2.86)	1,610 (33.62)	46 (1.76)	1,664 (38.96)
Unable to sit	93 (7.96)	620 (34.66)	11 (0.92)	801 (40.01)	56 (1.76)	1,604 (33.49)	22 (0.84)	1,659 (38.84)
Unable to use hands e.g. to feed	126 (11.01)	645 (36.05)	16 (1.35)	814 (40.66)	89 (2.83)	1,643 (34.31)	55 (2.13)	1,693 (39.64)
Vision								
Any vision problems including squint	352 (19.68) (30.99)	653 (36.5)	138 (6.89)	827 (41.31)	404 (12.98)	1,677 (35.02)	238 (9.34)	1,724 (40.37)
A not fully correctable visual defect	116 (10.4)	702 (39.24)	27 (2.3)	849 (42.41)	63 (2.07)	1,743 (36.40)	35 (1.40)	1,765 (41.33)
Complete blindness or able to see light only	26 (2.33)	673 (37.62)	0 (0) (0)	834 (41.66)	11 (0.36)	1,704 (35.58)	6 (0.24)	1,749 (40.95)
Hearing								
A hearing impairment	94 (8.37)	666 (37.23)	43 (3.72)	845 (42.21)	126 (4.08)	1,703 (35.56)	119 (4.72)	1,750 (40.97)
Hearing impairment correctable with aids	29 (2.61)	678 (37.90)	20 (1.73)	843 (42.11)	47 (1.52)	1,697 (35.44)	58 (2.29)	1,742 (40.79)
Hearing impairment not correctable with aids	23 (2.07)	679 (37.95)	6 (0.52)	849 (42.41)	21 (0.68)	1,720 (35.92)	23 (0.91)	1,752 (41.02)

© 2024 Rees P et al. *JAMA Network Open*.

eTable 4: Survival without severe neurodevelopmental impairment (NDI) and any NDI in survivors by each grade of IVH and laterality/extent of injury (adjusted covariates as per the low and high-grade IVH models; predictors of missingness also as per high and low-grade IVH models)					
Outcome	Crude OR (95%CI)	Adjusted OR (95%CI)	Absolute imputed risk for cases, n (%)	Absolute imputed risk for controls, n (%)	Imputed adjusted OR (95%CI)
IVH grade 1 vs. matched controls					
Survival without severe NDI	1.13 (0.98, 1.30)	1.05 (0.89, 1.25)	1,741 (68.96)	1,492 (66.95)	1.04 (0.88, 1.22)
Any NDI in survivors	1.01 (0.88, 1.17)	1.01 (0.86, 1.20)	967 (43.48)	820 (43.36)	1.02 (0.87, 1.19)
IVH grade2 vs. matched controls					
Survival without severe NDI	0.89 (0.78, 1.02)	0.76 (0.65, 0.90)	1,567 (59.70)	1,409 (63.33)	0.75 (0.64. 0.89)
Any NDI in survivors	1.30 (1.12, 1.50)	1.39 (1.17, 1.66)	1,141 (52.50)	828 (45.58)	1.38 (1.16, 1.64)
IVH grade 3 vs. matched controls					
Survival without severe NDI	0.56 (0.45, 0.69)	0.44 (0.33, 0.57)	417 (45.06)	486 (60.37)	0.44 (0.34, 0.57)
Any NDI in survivors	1.86 (1.43, 2.41)	1.94 (1.41, 2.66)	421 (63.07)	295 (47.25)	2.03 (1.49, 2.75)
IVH grade 4 vs. matched controls					
Survival without severe NDI	0.24 (0.20, 0.29)	0.18 (0.15, 0.23)	410 (24.43)	773 (58.83)	0.18 (0.15, 0.23)
Any NDI in survivors	5.24 (4.09, 6.72)	6.08 (4.53, 8.15)	746 (82.56)	473 (46.68)	6.29 (4.68, 8.44)
Bilateral low-grade IVH[a] vs. matched controls					
Survival without severe NDI	0.88 (0.74, 1.05)	0.82 (0.66, 1.02)	910 (60.49)	804 (62.67)	0.84 (0.68, 1.03)
Any NDI in survivors	1.56 (1.28, 1.89)	1.52 (1.20, 1.94)	668 (52.90)	446 (43.22)	1.53 (1.20, 1.94)
Unilateral low-grade[a] vs. matched controls					
Survival without severe NDI	1.30 (1.08, 1.55)	1.22 (0.98, 1.53)	1,037(70.58)	858 (65.78)	1.20 (0.96. 1.48)
Any NDI in survivors	0.94 (0.78, 1.13)	0.95 (0.76, 1.19)	557 (42.74)	484 (44.22)	0.97 (0.77 ,1.24)
Bilateral high-grade[b] vs. matched controls					
Survival without severe NDI	0.34 (0.28, 0.41)	0.27 (0.21, 0.34)	421 (33.43)	634 (59.99)	0.27 (0.21, 0.35)
Any NDI in survivors	3.44 (2.69, 4.40)	3.83 (2.86, 5.12)	618 (75.32)	377 (46.28)	3.97 (2.97, 5.30)
Unilateral high-grade[b] vs. matched controls					
Survival without severe NDI	0.79 (0.47, 1.12)	0.72 (0.42, 1.25)	130 (54.52)	116 (57.89)	0.90 (0.56, 1.47)
Any NDI in survivors	2.20 (1.33, 3.55)	2.49 (1.29, 4.65)	128 (66.30)	82 (48.55)	2.40 (1.32, 4.36)

a: 3,885 (70.4%) infants with low-grade IVH had laterality data available for analysis

b: 2,118 (75.4%) infants with high-grade IVH had laterality data available for analysis

© 2024 Rees P et al. *JAMA Network Open*.

eTable 5: Survival without severe neurodevelopmental impairment by week of gestation after high-grade IVH compared to controls								
Gestation	High grade IVH, n (%)	Controls, n (%)	Missing data, n (%)	Crude OR (95%CI)	Adjusted OR[a] (95%CI)	Absolute imputed risk after high-grade IVH, n (%)	Absolute imputed risk for controls, n (%)	Imputed adjusted OR (95%CI) [b]
22 (n=50)	1 (4.76)	5 (25.00)	9 (18)	-	-	-	-	-
23(n=804)	40 ((11.02)	84 (27.18)	132 (16.42)	0.33 (0.22, 0.50)	0.29 (0.17, 0.48)	51 (12.81)	110 (30.39)	0.31 (0.20, 0.49)
24 (n=1,169)	104 (20.12)	196 (47.00)	235 (20.10)	0.28 (0.21, 0.38)	0.23 (0.16, 0.33)	128 (21.83)	258 (50.72)	0.23 (0.16, 0.32)
25 (n=1,154)	154 (32.77)	208 (61.90)	348 (30.16)	0.30 (22, 0.40)	0.29 (0.21, 0.41)	201 (35.31)	298 (64.02)	0.30 (0.22, 0.42)
26 (n=904)	149 (41.62)	190 (68.84)	270 (29.87)	0.32 (0.23, 0.45)	0.29 (0.20, 0.43)	199 (43.91)	261 (70.52)	0.29 (0.19, 0.46)
27 (n=707)	123 (48.24)	187 (80.26)	219 (30.98)	0.23 (0.15, 0.34)	0.20 (0.13, 0.33)	163 (50.08)	251 (79.98)	0.23 (0.15, 0.36)
28 (n=484)	99 (43.23)	82 (75.23)	146 (30.17)	0.25 (0.15, 0.42)	0.21 (0.11, 0.39)	122 (44.51)	122 (75.82)	0.21 (0.11, 0.39)

[a] Adjusted for gestation, birthweight Z score, antenatal steroids, sex, index of median deprivation quintile, smoking in pregnancy and admission neonatal operational delivery network fitted as a random effect

[b] Predictors of missingness used in imputed models: maternal age, index of median deprivation quintile, birth year, gestation weeks, birthweight Z score, multimorbidity, smoking in pregnancy, discharge unit level, number of previous pregnancies, breastmilk at discharge and admission neonatal operational delivery network

© 2024 Rees P et al. *JAMA Network Open*.

eTable 6: the prevalence of additional morbidities for those with high-grade IVH, low-grade IVH and their matched controls				
Multimorbidity	High-grade, n (%)	Controls, n (%)	Low-grade, n (%)	Controls, n (%)
Severe retinopathy of prematurity*	59 (3.14)	32 (1.55)	70 (1.42)	35 (0.8)
Respiratory support at 36 weeks*	1,110 (59.14)	1,134 (54.97)	2,756 (56.04)	2,104 (48.12)
Surgically treated necrotizing enterocolitis*	196 (10.44)	91 (4.41)	283 (5.75)	167 (3.82)
Receipt of major surgery	741 (26.4)	418 (16.7)	1,043 (18.9)	748 (14.88)
Culture positive sepsis	598 (21.29)	273 (10.89)	1,070 (19.39)	414 (8.24)
Post-hemorrhagic ventricular dilatation	366 (13.03)	-	36 (0.65)	-
Ventriculoperitoneal shunt	132 (4.7)	-	47 (0.85)	-
Cystic periventricular leucomalacia	331 (11.78)	-	188 (3.41)	-
Seizures	360 (12.84)	-	209 (3.8)	-
Perinatal stroke	5 (0.18)	-	9 (0.16)	-
Central nervous system infection	173 (3.25)	-	152 (2.75)	-

*Major morbidities included in sub-group analysis of those who survived to 36 weeks' gestation

© 2024 Rees P et al. *JAMA Network Open*.

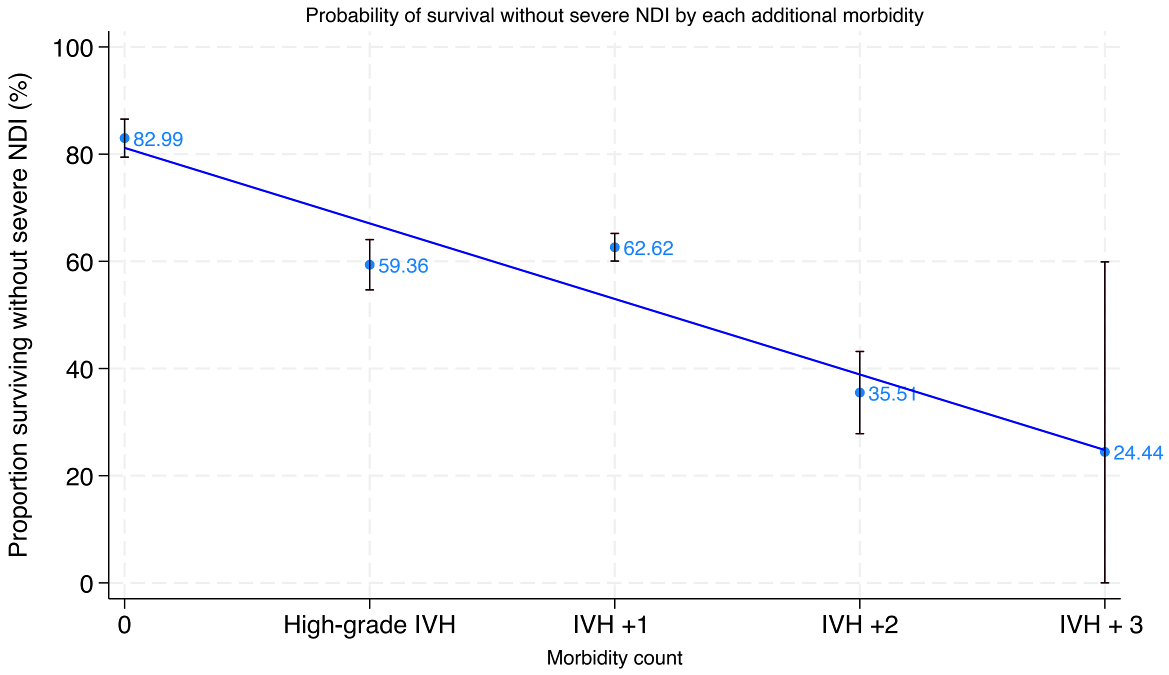

eFigure 3: graph illustrating the proportion of infants surviving without severe neurodevelopmental impairment (NDI) by increasing morbidity count

© 2024 Rees P et al. *JAMA Network Open*.

eTable 7: Estimated risk of survival without severe neurodevelopmental impairment under different missing at random (MAR) and missing not at random (MNAR) scenarios				
	Delta	Exponential (delta)	High-grade IVH aOR (95%CI)	Low-grade IVH aOR (95%CI)
Crude			0.33 (0.29, 0.38)	1.00 (0.91, 1.10)
MAR scenario	0	1	0.26 (0.22, 0.31)	0.90 (0.80, 1.01)
MNAR scenario	-0.4	0.6	0.27 (0.23, 0.32)	0.89 (0.79, 0.99)
	-0.3	0.7	0.27 (0.22, 0.32)	0.89 (0.79, 0.99)
	-0.2	0.8	0.27 (0.22, 0.32)	0.88 (0.79, 0.99)
	-0.1	0.9	0.26 (0.22, 0.31)	0.88 (0.79, 0.99)
	0.1	1.1	0.26 (0.22, 0.31)	0.88 (0.79, 0.99)
	0.2	1.2	0.26 (0.22, 0.31)	0.88 (0.79, 0.99)

© 2024 Rees P et al. *JAMA Network Open*.

eTable 8: absolute imputed risk of survival without severe neurodevelopmental impairment under the missing at random (MAR) assumption and moderate missing not at random (MNAR) scenario				
	Absolute imputed risk in high-grade IVH, n (%)	Absolute imputed risk in matched controls, n (%)	Absolute imputed risk in low-grade IVH, n (%)	Absolute imputed risk in matched controls, n (%)
MAR (delta 0)	861 (32.77)	1342 (59.78)	3,290 (64.41)	2,908 (65.19)
MNAR (delta 0.6)	827 (31.47)	1307(58.00)	3,205 (62.74)	2,834 (63.52)

© 2024 Rees P et al. *JAMA Network Open*.
