# Supplementary material for: Intraventricular Hemorrhage and Survival, Multimorbidity, and Neurodevelopment
Source: JAMA Netw Open. 2025 Jan 6;8(1):e2452883. doi: 10.1001/jamanetworkopen.2024.52883 (PMC11704976; doi:10.1001/jamanetworkopen.2024.52883)
Supplement: Supplement 2. — Data Sharing Statement [file jamanetwopen-e2452883-s002.pdf]

Data Sharing Statement

Rees. Trends in Intraventricular Hemorrhage and Survival, Multimorbidity, and Neurodevelopment. *JAMA Netw Open*. Published January 02, 2025. doi:10.1001/jamanetworkopen.2024.52883

Data
Data available: No
